# 1,3-Bis(4-methyl­benz­yl)pyrimidine-2,4(1*H*,3*H*)-dione

**DOI:** 10.1107/S1600536809053586

**Published:** 2009-12-19

**Authors:** Gong-Chun Li, Xiao-Ping Song, Li-Ke Zhang, Jiao-Jiao Cui, Feng-Ling Yang

**Affiliations:** a College of Chemistry and Chemical Engineering, Xuchang University, Xuchang, Henan Province 461000, People’s Republic of China

## Abstract

In the title mol­ecule, C_20_H_20_N_2_O_2_, the central pyrimidine ring forms dihedral angles of 71.9 (1) and 69.8 (1)° with the two benzene rings. In the crystal, weak inter­molecular C—H⋯O hydrogen bonds link mol­ecules into centrosymmetric dimers. The crystal packing exhibits also π–π inter­actions as indicated by short distances of 3.674 (2) Å between the centroids of the pyrimidine rings of neighbouring mol­ecules.

## Related literature

For the crystal structure of 1,3-bis­(4-chloro­benz­yl)pyrimidine-2,4(1*H*,3*H*)-dione, see: Yang & Li (2006 ▶).
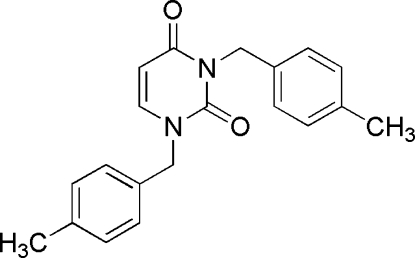


## Experimental

### 

#### Crystal data

C_20_H_20_N_2_O_2_
*M* *_r_* = 320.38
Triclinic, 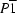

*a* = 9.4182 (19) Å
*b* = 10.102 (2) Å
*c* = 10.448 (2) Å
α = 66.25 (3)°
β = 80.79 (3)°
γ = 71.18 (3)°
*V* = 860.7 (3) Å^3^
*Z* = 2
Mo *K*α radiation
μ = 0.08 mm^−1^
*T* = 293 K
0.20 × 0.20 × 0.20 mm

#### Data collection

Rigaku Saturn CCD area-detector diffractometer
Absorption correction: multi-scan (*CrystalClear*; Rigaku/MSC, 2006 ▶) *T*_min_ = 0.984, *T*_max_ = 0.984
8622 measured reflections
3001 independent reflections
2529 reflections with *I* > 2σ(*I*)
*R*_int_ = 0.034

#### Refinement

*R*[*F* ^2^ > 2σ(*F* ^2^)] = 0.088
*wR*(*F* ^2^) = 0.161
*S* = 1.27
3001 reflections
219 parameters
H-atom parameters constrained
Δρ_max_ = 0.14 e Å^−3^
Δρ_min_ = −0.17 e Å^−3^

### 

Data collection: *CrystalClear* (Rigaku/MSC, 2006 ▶); cell refinement: *CrystalClear*; data reduction: *CrystalClear*; program(s) used to solve structure: *SHELXS97* (Sheldrick, 2008 ▶); program(s) used to refine structure: *SHELXL97* (Sheldrick, 2008 ▶); molecular graphics: *SHELXTL* (Sheldrick, 2008 ▶); software used to prepare material for publication: *SHELXTL*.

## Supplementary Material

Crystal structure: contains datablocks global, I. DOI: 10.1107/S1600536809053586/cv2673sup1.cif

Structure factors: contains datablocks I. DOI: 10.1107/S1600536809053586/cv2673Isup2.hkl

Additional supplementary materials:  crystallographic information; 3D view; checkCIF report

## Figures and Tables

**Table 1:** Hydrogen-bond geometry (Å, °)

*D*—H⋯*A*	*D*—H	H⋯*A*	*D*⋯*A*	*D*—H⋯*A*
C14—H14⋯O2^i^	0.93	2.50	3.430 (4)	174

Symmetry code: (i) 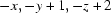.

**Fig. 1.**
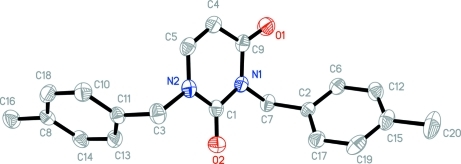
The molecular structure of (I), with displacement ellipsoids drawn at the 30% probability level. H atoms omitted for clarity.

## supplementary crystallographic information

### Comment

In continuation of our search for new biologically active pyrimidine derivatives (Yang & Li, 2006), we present here the title compound (I).

In (I) (Fig. 1), all bond lengths and angles are normal and correspond to those observed in the related 1,3-bis(4-chlorobenzyl)pyrimidine-2,4(1*H*,3*H*)-dione (Yang & Li, 2006). The central pyrimidine ring forms dihedral angles of 71.9 (1)° and 69.8 (1)° with the two benzene rings, respectively. Weak intermolecular C—H···O hydrogen bonds (Table 1) link molecules into centrosymmetric dimers. The crystal packing exhibits also π-π interactions proved by short distances of 3.674 (2) Å between the centroids of pyrimidine rings from the neighbouring molecules.

### Experimental

Uracil (0.56 g, 5 mmol) and anhydrous potassium carbonate (0.84 g, 6 mmol) were mixed in *N*,*N*-dimethylformamide (20 ml). A solution of 4-methyl-benzyl chloride (0.70 g, 5 mmol) in acetone (10 ml) was then added dropwise, with stirring, at room temperature, and the mixture was stirred for another 10 h and then refluxed for 4 h. The solvent was evaporated *in vacuo* and the residue was washed with water. The resulting white precipitate was filtered off and purified by column chromatography on silica gel (petroleum ether:ethyl acetate = 2:1). The title compound was recrystallized from ethanol and single crystals of (I) were obtained by slow evaporation.

### Refinement

All H atoms were placed in calculated positions, with C—H = 0.93 - 0.97 Å, and included in the final cycles of refinement using a riding model, with *U*_iso_(H) = 1.2–1.5 *U_eq_*(C).

### Crystal data

**Table:**

C_20_H_20_N_2_O_2_	*Z* = 2
*M**_r_* = 320.38	*F*(000) = 340
Triclinic, *P*1	*D*_x_ = 1.236 Mg m^−^^3^
*a* = 9.4182 (19) Å	Mo *K*α radiation, λ = 0.71073 Å
*b* = 10.102 (2) Å	Cell parameters from 2459 reflections
*c* = 10.448 (2) Å	θ = 2.1–27.9°
α = 66.25 (3)°	µ = 0.08 mm^−^^1^
β = 80.79 (3)°	*T* = 293 K
γ = 71.18 (3)°	Prism, colourless
*V* = 860.7 (3) Å^3^	0.20 × 0.20 × 0.20 mm

### Data collection

**Table:**

Rigaku Saturn CCD area-detector diffractometer	3001 independent reflections
Radiation source: fine-focus sealed tube	2529 reflections with *I* > 2σ(*I*)
graphite	*R*_int_ = 0.034
Detector resolution: 28.5714 pixels mm^-1^	θ_max_ = 25.0°, θ_min_ = 2.1°
phi and ω scans	*h* = −11→11
Absorption correction: multi-scan (*CrystalClear*; Rigaku/MSC, 2006)	*k* = −11→12
*T*_min_ = 0.984, *T*_max_ = 0.984	*l* = −12→12
8622 measured reflections	

### Refinement

**Table:**

Refinement on *F*^2^	Secondary atom site location: difference Fourier map
Least-squares matrix: full	Hydrogen site location: inferred from neighbouring sites
*R*[*F*^2^ > 2σ(*F*^2^)] = 0.088	H-atom parameters constrained
*wR*(*F*^2^) = 0.161	*w* = 1/[σ^2^(*F*_o_^2^) + (0.0409*P*)^2^ + 0.3734*P*] where *P* = (*F*_o_^2^ + 2*F*_c_^2^)/3
*S* = 1.27	(Δ/σ)_max_ < 0.001
3001 reflections	Δρ_max_ = 0.14 e Å^−^^3^
219 parameters	Δρ_min_ = −0.17 e Å^−^^3^
Primary atom site location: structure-invariant direct methods	

### Special details

**Table:**

Experimental. Software + citation
Geometry. All e.s.d.'s (except the e.s.d. in the dihedral angle between two l.s. planes) are estimated using the full covariance matrix. The cell e.s.d.'s are taken into account individually in the estimation of e.s.d.'s in distances, angles and torsion angles; correlations between e.s.d.'s in cell parameters are only used when they are defined by crystal symmetry. An approximate (isotropic) treatment of cell e.s.d.'s is used for estimating e.s.d.'s involving l.s. planes.
Refinement. Refinement of *F*^2^ against ALL reflections. The weighted *R*-factor *wR* and goodness of fit *S* are based on *F*^2^, conventional *R*-factors *R* are based on *F*, with *F* set to zero for negative *F*^2^. The threshold expression of *F*^2^ > σ(*F*^2^) is used only for calculating *R*-factors(gt) *etc*. and is not relevant to the choice of reflections for refinement. *R*-factors based on *F*^2^ are statistically about twice as large as those based on *F*, and *R*- factors based on ALL data will be even larger.

### Fractional atomic coordinates and isotropic or equivalent isotropic displacement parameters (Å^2^)

**Table:**

	*x*	*y*	*z*	*U*_iso_*/*U*_eq_	
O1	0.4982 (3)	0.1871 (3)	0.6026 (3)	0.0711 (7)	
O2	0.3392 (2)	0.4909 (3)	0.8542 (2)	0.0661 (7)	
N1	0.4156 (2)	0.3369 (3)	0.7299 (2)	0.0452 (6)	
N2	0.2678 (3)	0.5867 (3)	0.6284 (3)	0.0491 (6)	
C1	0.3407 (3)	0.4728 (3)	0.7458 (3)	0.0483 (7)	
C2	0.6580 (3)	0.1972 (3)	0.8500 (3)	0.0431 (7)	
C3	0.1828 (3)	0.7346 (3)	0.6372 (4)	0.0596 (9)	
H3A	0.1960	0.8147	0.5494	0.072*	
H3B	0.2227	0.7467	0.7105	0.072*	
C4	0.3402 (3)	0.4340 (4)	0.4937 (3)	0.0562 (8)	
H4	0.3370	0.4235	0.4099	0.067*	
C5	0.2677 (3)	0.5635 (4)	0.5079 (3)	0.0543 (8)	
H5	0.2143	0.6422	0.4330	0.065*	
C6	0.7652 (3)	0.0843 (3)	0.8165 (3)	0.0529 (8)	
H6	0.7358	0.0149	0.7973	0.064*	
C7	0.4931 (3)	0.2105 (3)	0.8525 (3)	0.0521 (8)	
H7A	0.4827	0.1170	0.8550	0.063*	
H7B	0.4451	0.2249	0.9372	0.063*	
C8	−0.2911 (3)	0.7765 (3)	0.7206 (3)	0.0492 (8)	
C9	0.4242 (3)	0.3092 (4)	0.6070 (3)	0.0526 (8)	
C10	−0.0848 (4)	0.8346 (4)	0.5629 (3)	0.0577 (8)	
H10	−0.0510	0.8844	0.4730	0.069*	
C11	0.0171 (3)	0.7490 (3)	0.6673 (3)	0.0473 (7)	
C12	0.9150 (4)	0.0739 (4)	0.8113 (3)	0.0614 (9)	
H12	0.9854	−0.0040	0.7901	0.074*	
C13	−0.0374 (4)	0.6768 (4)	0.7999 (3)	0.0599 (9)	
H13	0.0286	0.6189	0.8726	0.072*	
C14	−0.1891 (4)	0.6899 (3)	0.8254 (3)	0.0582 (9)	
H14	−0.2231	0.6392	0.9150	0.070*	
C15	0.9639 (4)	0.1749 (4)	0.8365 (3)	0.0630 (10)	
C16	−0.4572 (3)	0.7930 (4)	0.7502 (4)	0.0696 (10)	
H16A	−0.5029	0.8770	0.7787	0.104*	
H16B	−0.4712	0.7025	0.8235	0.104*	
H16C	−0.5030	0.8099	0.6671	0.104*	
C17	0.7049 (4)	0.2967 (4)	0.8803 (3)	0.0601 (9)	
H17	0.6349	0.3714	0.9064	0.072*	
C18	−0.2361 (4)	0.8480 (4)	0.5894 (4)	0.0610 (9)	
H18	−0.3022	0.9066	0.5168	0.073*	
C19	0.8569 (4)	0.2856 (4)	0.8720 (4)	0.0717 (11)	
H19	0.8869	0.3548	0.8909	0.086*	
C20	1.1291 (4)	0.1673 (6)	0.8239 (4)	0.1041 (16)	
H20A	1.1884	0.0636	0.8601	0.156*	
H20B	1.1462	0.2195	0.8764	0.156*	
H20C	1.1572	0.2137	0.7274	0.156*	

### Atomic displacement parameters (Å^2^)

**Table:**

	*U*^11^	*U*^22^	*U*^33^	*U*^12^	*U*^13^	*U*^23^
O1	0.0713 (16)	0.0577 (15)	0.0837 (18)	−0.0011 (12)	−0.0192 (13)	−0.0334 (13)
O2	0.0646 (15)	0.0773 (16)	0.0552 (14)	−0.0105 (12)	−0.0044 (11)	−0.0303 (13)
N1	0.0379 (13)	0.0480 (15)	0.0434 (14)	−0.0112 (11)	−0.0041 (11)	−0.0106 (12)
N2	0.0424 (14)	0.0438 (14)	0.0534 (16)	−0.0082 (11)	−0.0015 (12)	−0.0138 (12)
C1	0.0381 (17)	0.0513 (19)	0.052 (2)	−0.0148 (14)	0.0023 (14)	−0.0162 (16)
C2	0.0462 (17)	0.0411 (16)	0.0355 (15)	−0.0103 (14)	−0.0092 (13)	−0.0066 (13)
C3	0.0519 (19)	0.0480 (19)	0.076 (2)	−0.0136 (16)	0.0004 (16)	−0.0216 (17)
C4	0.053 (2)	0.057 (2)	0.058 (2)	−0.0112 (17)	−0.0080 (16)	−0.0218 (17)
C5	0.0481 (19)	0.058 (2)	0.0452 (19)	−0.0149 (16)	−0.0077 (14)	−0.0052 (16)
C6	0.055 (2)	0.0476 (18)	0.0524 (19)	−0.0145 (15)	−0.0082 (15)	−0.0126 (15)
C7	0.0487 (18)	0.0488 (18)	0.0469 (18)	−0.0157 (15)	−0.0059 (14)	−0.0032 (14)
C8	0.0509 (19)	0.0464 (18)	0.0543 (19)	−0.0124 (15)	0.0041 (15)	−0.0262 (15)
C9	0.0415 (18)	0.054 (2)	0.062 (2)	−0.0143 (16)	−0.0056 (15)	−0.0203 (17)
C10	0.059 (2)	0.056 (2)	0.0474 (19)	−0.0160 (16)	0.0000 (15)	−0.0094 (16)
C11	0.0463 (17)	0.0376 (16)	0.0568 (19)	−0.0090 (14)	−0.0008 (15)	−0.0192 (14)
C12	0.052 (2)	0.060 (2)	0.054 (2)	−0.0038 (17)	−0.0051 (16)	−0.0103 (17)
C13	0.056 (2)	0.060 (2)	0.0489 (19)	−0.0025 (16)	−0.0081 (16)	−0.0143 (16)
C14	0.060 (2)	0.054 (2)	0.0499 (19)	−0.0094 (17)	0.0092 (16)	−0.0181 (16)
C15	0.050 (2)	0.073 (2)	0.047 (2)	−0.0214 (19)	−0.0100 (15)	0.0015 (17)
C16	0.053 (2)	0.081 (3)	0.083 (3)	−0.0202 (18)	0.0078 (18)	−0.042 (2)
C17	0.058 (2)	0.056 (2)	0.068 (2)	−0.0105 (17)	−0.0118 (17)	−0.0254 (17)
C18	0.051 (2)	0.070 (2)	0.057 (2)	−0.0112 (17)	−0.0121 (16)	−0.0191 (18)
C19	0.075 (3)	0.072 (2)	0.079 (3)	−0.034 (2)	−0.024 (2)	−0.021 (2)
C20	0.055 (2)	0.149 (4)	0.084 (3)	−0.042 (3)	−0.010 (2)	−0.007 (3)

### Geometric parameters (Å, °)

**Table:**

O1—C9	1.221 (4)	C8—C14	1.382 (4)
O2—C1	1.215 (3)	C8—C16	1.513 (4)
N1—C1	1.390 (4)	C10—C18	1.381 (4)
N1—C9	1.405 (4)	C10—C11	1.378 (4)
N1—C7	1.478 (3)	C10—H10	0.9300
N2—C5	1.370 (4)	C11—C13	1.383 (4)
N2—C1	1.386 (4)	C12—C15	1.371 (5)
N2—C3	1.482 (4)	C12—H12	0.9300
C2—C17	1.376 (4)	C13—C14	1.383 (4)
C2—C6	1.381 (4)	C13—H13	0.9300
C2—C7	1.512 (4)	C14—H14	0.9300
C3—C11	1.512 (4)	C15—C19	1.375 (5)
C3—H3A	0.9700	C15—C20	1.520 (4)
C3—H3B	0.9700	C16—H16A	0.9600
C4—C5	1.322 (4)	C16—H16B	0.9600
C4—C9	1.439 (4)	C16—H16C	0.9600
C4—H4	0.9300	C17—C19	1.391 (4)
C5—H5	0.9300	C17—H17	0.9300
C6—C12	1.375 (4)	C18—H18	0.9300
C6—H6	0.9300	C19—H19	0.9300
C7—H7A	0.9700	C20—H20A	0.9600
C7—H7B	0.9700	C20—H20B	0.9600
C8—C18	1.374 (4)	C20—H20C	0.9600
			
C1—N1—C9	125.6 (3)	C18—C10—C11	121.3 (3)
C1—N1—C7	117.3 (3)	C18—C10—H10	119.4
C9—N1—C7	117.1 (3)	C11—C10—H10	119.4
C5—N2—C1	121.7 (3)	C10—C11—C13	117.7 (3)
C5—N2—C3	119.8 (3)	C10—C11—C3	120.8 (3)
C1—N2—C3	118.5 (3)	C13—C11—C3	121.5 (3)
O2—C1—N2	122.4 (3)	C6—C12—C15	122.0 (3)
O2—C1—N1	122.7 (3)	C6—C12—H12	119.0
N2—C1—N1	114.9 (3)	C15—C12—H12	119.0
C17—C2—C6	118.5 (3)	C14—C13—C11	120.7 (3)
C17—C2—C7	121.2 (3)	C14—C13—H13	119.6
C6—C2—C7	120.3 (3)	C11—C13—H13	119.6
N2—C3—C11	111.9 (2)	C8—C14—C13	121.5 (3)
N2—C3—H3A	109.2	C8—C14—H14	119.3
C11—C3—H3A	109.2	C13—C14—H14	119.3
N2—C3—H3B	109.2	C12—C15—C19	117.3 (3)
C11—C3—H3B	109.2	C12—C15—C20	121.7 (4)
H3A—C3—H3B	107.9	C19—C15—C20	120.9 (4)
C5—C4—C9	120.5 (3)	C8—C16—H16A	109.5
C5—C4—H4	119.8	C8—C16—H16B	109.5
C9—C4—H4	119.8	H16A—C16—H16B	109.5
C4—C5—N2	122.8 (3)	C8—C16—H16C	109.5
C4—C5—H5	118.6	H16A—C16—H16C	109.5
N2—C5—H5	118.6	H16B—C16—H16C	109.5
C12—C6—C2	120.5 (3)	C2—C17—C19	120.1 (3)
C12—C6—H6	119.8	C2—C17—H17	120.0
C2—C6—H6	119.8	C19—C17—H17	120.0
N1—C7—C2	112.6 (2)	C10—C18—C8	121.4 (3)
N1—C7—H7A	109.1	C10—C18—H18	119.3
C2—C7—H7A	109.1	C8—C18—H18	119.3
N1—C7—H7B	109.1	C15—C19—C17	121.6 (3)
C2—C7—H7B	109.1	C15—C19—H19	119.2
H7A—C7—H7B	107.8	C17—C19—H19	119.2
C18—C8—C14	117.5 (3)	C15—C20—H20A	109.5
C18—C8—C16	121.4 (3)	C15—C20—H20B	109.5
C14—C8—C16	121.1 (3)	H20A—C20—H20B	109.5
O1—C9—N1	120.2 (3)	C15—C20—H20C	109.5
O1—C9—C4	125.3 (3)	H20A—C20—H20C	109.5
N1—C9—C4	114.4 (3)	H20B—C20—H20C	109.5
			
C5—N2—C1—O2	−178.4 (3)	C5—C4—C9—O1	−177.7 (3)
C3—N2—C1—O2	−1.2 (4)	C5—C4—C9—N1	2.5 (4)
C5—N2—C1—N1	1.6 (4)	C18—C10—C11—C13	−0.1 (5)
C3—N2—C1—N1	178.9 (2)	C18—C10—C11—C3	179.8 (3)
C9—N1—C1—O2	−178.8 (3)	N2—C3—C11—C10	−103.7 (3)
C7—N1—C1—O2	1.1 (4)	N2—C3—C11—C13	76.1 (4)
C9—N1—C1—N2	1.2 (4)	C2—C6—C12—C15	−1.1 (5)
C7—N1—C1—N2	−178.9 (2)	C10—C11—C13—C14	0.5 (5)
C5—N2—C3—C11	80.2 (3)	C3—C11—C13—C14	−179.3 (3)
C1—N2—C3—C11	−97.2 (3)	C18—C8—C14—C13	0.8 (5)
C9—C4—C5—N2	0.0 (5)	C16—C8—C14—C13	−178.6 (3)
C1—N2—C5—C4	−2.2 (4)	C11—C13—C14—C8	−0.9 (5)
C3—N2—C5—C4	−179.5 (3)	C6—C12—C15—C19	2.1 (5)
C17—C2—C6—C12	−1.1 (4)	C6—C12—C15—C20	−177.1 (3)
C7—C2—C6—C12	178.7 (3)	C6—C2—C17—C19	2.3 (5)
C1—N1—C7—C2	−95.8 (3)	C7—C2—C17—C19	−177.5 (3)
C9—N1—C7—C2	84.1 (3)	C11—C10—C18—C8	0.0 (5)
C17—C2—C7—N1	74.8 (4)	C14—C8—C18—C10	−0.3 (5)
C6—C2—C7—N1	−105.0 (3)	C16—C8—C18—C10	179.0 (3)
C1—N1—C9—O1	177.0 (3)	C12—C15—C19—C17	−0.9 (5)
C7—N1—C9—O1	−2.8 (4)	C20—C15—C19—C17	178.3 (3)
C1—N1—C9—C4	−3.2 (4)	C2—C17—C19—C15	−1.3 (5)
C7—N1—C9—C4	176.9 (2)		

### Hydrogen-bond geometry (Å, °)

**Table:**

*D*—H···*A*	*D*—H	H···*A*	*D*···*A*	*D*—H···*A*
C14—H14···O2^i^	0.93	2.50	3.430 (4)	174

Symmetry codes: (i) −*x*, −*y*+1, −*z*+2.
